# Tactile exploration and imagery elicit distinct neural dynamics in the parietal cortical network

**DOI:** 10.3389/fnins.2025.1621383

**Published:** 2025-07-24

**Authors:** Qi Zhang, Yang Yang, Zhemeng Wang, Jiayue Zhou, Runshi Gao, Xingyi Yang, Siwei Li, Tao Yu, Jin Zhou, Changyong Wang

**Affiliations:** ^1^School of Life Science and Technology, Harbin Institute of Technology, Harbin, China; ^2^Beijing Institute of Basic Medical Sciences, Beijing, China; ^3^School of Foreign Languages, Peking University, Beijing, China; ^4^School of Clinical Medicine, Capital Medical University, Beijing, China; ^5^Department of Neurosurgery, Beijing Institute of Functional Neurosurgery, Xuanwu Hospital, Capital Medical University, Beijing, China; ^6^Beijing Simulation Center, Beijing, China

**Keywords:** tactile imagery, somatosensory cortex, posterior parietal cortex, texture scanning, SEEG (stereo-electroencephalography)

## Abstract

**Background:**

Tactile imagery involves the reconstruction of sensory experiences without actual tactile input. While tactile perception and imagery exhibit similar spatial patterns of neural activation, the underlying neural dynamics, particularly cortical communications within the parietal network, remain unclear.

**Methods:**

The present study recruited 5 patients with implanted stereo-electroencephalography (sEEG) electrodes and recorded sEEG data during texture scanning and imagery. Local neural representations and interregional communications among parietal cortical regions were analyzed.

**Results:**

Opposing modulation patterns of local time-frequency representations were observed, with inhibited neural synchronization during texture scanning and activated synchronization during texture imagery. Consistently, the directional communication from the somatosensory cortex to the posterior parietal cortex (PPC) was found to be suppressed for scanning but enhanced for imagery. Additionally, bidirectional communication between the supramarginal gyrus and precuneus was activated during imagery but not scanning, suggesting a unique pathway for reconstructing tactile experiences.

**Conclusion:**

Our findings proposed that while texture perception and imagery engage overlapping cortical regions, their mechanisms underlying local encoding and interregional communication are distinct.

## 1 Introduction

Tactile sensation guides the interaction with the environment by modulating object manipulation in response to sensory feedback. For patients with sensorimotor disorders, artificial tactile sensations can be induced through microstimulation of the somatosensory cortex (Flesher et al., 2016; Greenspon et al., 2024; Hughes et al., 2021), enhancing neuroprosthetic control (Flesher et al., 2021; Valle et al., 2025). While tactile stimulation is encoded in the somatosensory cortex (Delhaye et al., 2018; Jiang et al., 2018; Lieber and Bensmaia, 2019, 2020; Long et al., 2022; Rossi-Pool et al., 2021), the mechanisms underlying its projection to higher-order brain regions for the formation of sensory perception remain unclear. In particular, for patients with impaired sensory inputs, understanding how tactile experience is generated without real sensory input is crucial for improving the effectiveness of artificial sensation.

Tactile imagery is a top-down process that reconstructs past sensory experiences, recruiting a distributed neural network including the somatosensory cortex and the posterior parietal cortex (Yoo et al., 2003). It provides insights into the formation of tactile experiences, particularly for patients whose sensory input functions are impaired (Bashford et al., 2021; Chivukula et al., 2021). Recent studies have highlighted the potential of tactile imagery in neurorehabilitation. It was demonstrated that tactile imagery achieved classification performance comparable to motor imagery in brain-computer interface (BCI) applications (Sengupta and Lakshminarayanan, 2024; Yao et al., 2018, 2022) and further enhanced motor decoding when integrated with motor imagery (Ahn et al., 2014; Zhong et al., 2023). Furthermore, prolonged training in tactile imagery was shown to enhance both BCI performance (Yao et al., 2019) and cognitive function (Lakshminarayanan et al., 2023), suggesting that tactile imagery would be a promising strategy for cognitive and motor rehabilitation.

There has been a debate of whether perceived and imagined tactile sensation share common neural functions. Neuroimaging studies found that tactile imagery evoked somatotopic activation alike actual perception within the somatosensory cortex, supporting the existence of common neural substrates (Nierhaus et al., 2023; Schmidt et al., 2014; Schmidt and Blankenburg, 2019; Yoo et al., 2003). Similarly, electroencephalography (EEG) studies have demonstrated contralateral event-related desynchronization (ERD) in the somatosensory cortex during tactile imagery, mirroring the neural responses elicited by actual tactile stimuli (Morozova et al., 2024; Wen et al., 2024; Yakovlev et al., 2023). However, some findings suggest that while tactile perception and imagery share common locations of neural activities, the directionality of neural projection is reversed (Dentico et al., 2014). Specifically, tactile perception with actual input involves a bottom-up process storing sensory information in higher-order regions, whereas tactile imagery reinstates past sensory experiences in the somatosensory cortex via top-down processing.

The posterior parietal cortex (PPC) has been recognized for its role in multisensory integration and sensorimotor coordination. Functionally interconnected with multiple brain regions, including the sensory cortex, the PPC integrates multimodal sensory inputs to construct coherent perceptual representations, which are then utilized to regulate motion through sensory feedback (Akrami et al., 2018; Creem-Regehr, 2009; Delhaye et al., 2018; Klautke et al., 2023; Whitlock, 2017). PPC has been shown to encode somatotopic tactile perception (Huang et al., 2012; Sereno and Huang, 2014) and respond to tactile properties such as object size and shape during sensorimotor interactions (Michaels et al., 2020; Schaffelhofer et al., 2015). Recent studies suggest that the PPC is also involved in tactile cognitive processes that occur in the absence of real sensory input. In clinical studies involving patients implanted with microelectrode arrays, the supramarginal gyrus (SMG) and the junction of the intraparietal sulcus and postcentral sulcus (PC-IP) have been found to encode tactile information during observation and imagery of touching (Bashford et al., 2021; Chivukula et al., 2021, 2025). It was also found that tactile imagery elicited body-part-specific responses locally in PPC similar to actual touch, supporting the hypothesis of overlapping neural mechanisms within higher-order cognitive centers. However, the neural pathway through which the somatosensory cortex transfers information to the PPC, as well as the internal communication dynamics within the PPC during tactile processing, remain unclear.

Investigating interregional communication of tactile imagery has been challenging, as neuroimaging approaches such as fMRI are constrained by limited temporal resolution, making it difficult to capture frequency-domain features, particularly in high-frequency bands. Stereo-electroencephalography (sEEG), which involves the implantation of electrodes with multiple contacts across different brain regions (Cardinale et al., 2016; Parvizi and Kastner, 2018), enables the simultaneous recording of neural activity across cortical and subcortical areas with both broad spatial coverage and high temporal resolution. It allows for the characterization of network connectivity within the parietal cortex in both the time and frequency domains. SEEG has been employed to decode various sensorimotor functions, including different hand postures, movements, and tactile sensations (Bouton et al., 2021; Breault et al., 2019; Li et al., 2022; Wu et al., 2022, 2023; Wu et al., 2024b), and has been applied in recovery of language disorders (Huang et al., 2021; Wu et al., 2024a). Despite its advantages, no studies to date have comprehensively investigated the parietal neural networks including the somatosensory cortex and PPC during tactile perception and imagery with sEEG.

The current study aimed to identify the local neural representation and interregional communication of the parietal cortical network in clinical patients implanted with sEEG electrodes. As manual texture perception typically involves active hand–environment interaction and can influence object manipulation strategies (Picard and Smith, 1992), an active texture perception task was employed to better capture task-relevant tactile processing. The findings revealed dissociable patterns of local neural synchronization and interregional connectivity within the parietal cortical network between texture scanning and imagery. These results suggested that although both tactile perception and imagery are encoded in somatosensory and posterior parietal cortices, they engage distinct patterns of neural responses and communications.

## 2 Materials and methods

### 2.1 Participants

Five participants were included in the current study (2 males, 3 females; mean age: 30 ± 8.78 years). All participants were patients with refractory epilepsy and were undergoing presurgical assessment. Participation was entirely voluntary, and all individuals were informed that their involvement in the study would not influence their clinical treatment and that they retained the right to withdraw at any time. Additionally, all experimental procedures were in accordance with the Declaration of Helsinki and were approved by the Ethics Committee of Xuanwu Hospital, Capital Medical University. Notably, all participants remained seizure-free throughout the data collection period.

### 2.2 Implantations

Electrode placement was determined exclusively based on clinical requirements and was independent of the study’s objectives. Each participant was implanted with 6 to 18 semi-rigid platinum/iridium electrodes (shaft diameter: 0.8 mm), with each electrode containing 8 to 16 recording contacts (contact length: 2 mm; inter-contact space: 1.5 mm; Huake Hengsheng, Beijing, China). Two participants received electrode implantation in the left hemisphere, two in the right hemisphere, and one underwent bilateral implantation. Across all five participants, a total of 850 recording contacts were implanted.

High-resolution pre-operative T1-weighted MPRAGE volumes were processed with FreeSurfer^1^ to generate subject-specific cortical segmentations in native space. Centroids of electrode contacts, localized on post-implantation CT scans, were rigidly coregistered to the MPRAGE volumes and projected onto the segmented cortical surface, thereby assigning each recording site an unambiguous neuroanatomical label. For group-level visualization, the resulting electrode coordinates were subsequently normalized to MNI-space via an affine transformation implemented in SPM12.^2^

**Figure 1:**
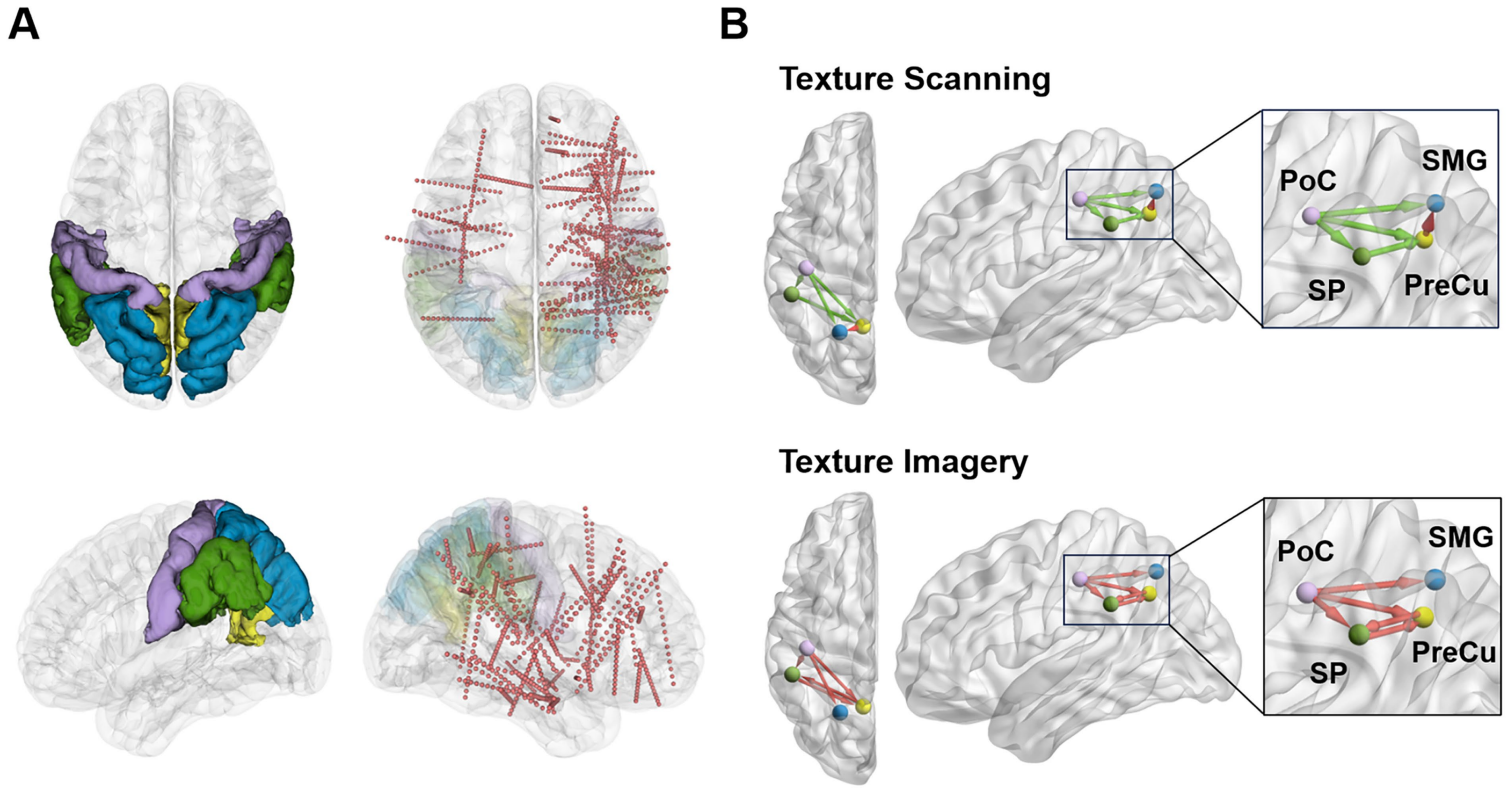
Schematic diagrams of implantation locations and associated parietal networks during texture scanning and imagery. **(A)** Contacts in parietal regions (PoC: purple, PreCu: yellow, SP: blue, SMG: green) were taken into analysis. The red dots represent sEEG contacts. The plots were generated with 3D Slicer version 5.6.2. **(B)** Overview of the dissociable parietal network patterns quantified by Granger causality during texture scanning and imagery. The plots were generated with BrainNet Viewer version 1.7 (Xia et al., 2013).

In accordance with the Desikan-Killiany cortical parcellation template (Desikan et al., 2006), the parietal cortex in this study was segmented into the inferior parietal cortex (IP), postcentral gyrus (PoC), precuneus cortex (PreCu), superior parietal cortex (SP), and supramarginal gyrus (SMG, Figure 1). Data from contacts located in PoC (*n* = 17 contacts), PreCu (*n* = 12), SP (*n* = 12), and SMG (*n* = 24) were included in the following analysis (see more details in Table 1). IP was excluded due to the limited number of recording contacts.

**Table 1:** Number of electrode contacts in each region of interest.

Participants	PoC	PreCu	SP	SMG
S1	4	2	1	6
S2	3	6	4	8
S3	0	3	4	3
S4	0	0	0	4
S5	10	1	3	3

### 2.3 Experimental paradigm and procedures

During the task, the patient reclined on a hospital bed with eyes directed toward a monitor positioned approximately 2 m away at the end of the bed. The hand contralateral to the electrode implantation site rested toward a circular rotating platform on the table. Four distinct textured materials—synthetic fur, paper, glass, and fabric—were mounted on the circular rotating platform, each covering one-quarter of its surface. A U-shaped barrier was positioned between the participant and the platform, restricting visual access but permitting tactile contact via the fingers. When a specific texture was to be explored, the operator remotely rotated the platform to align the corresponding material to the participant’s finger.

**Figure 2:**
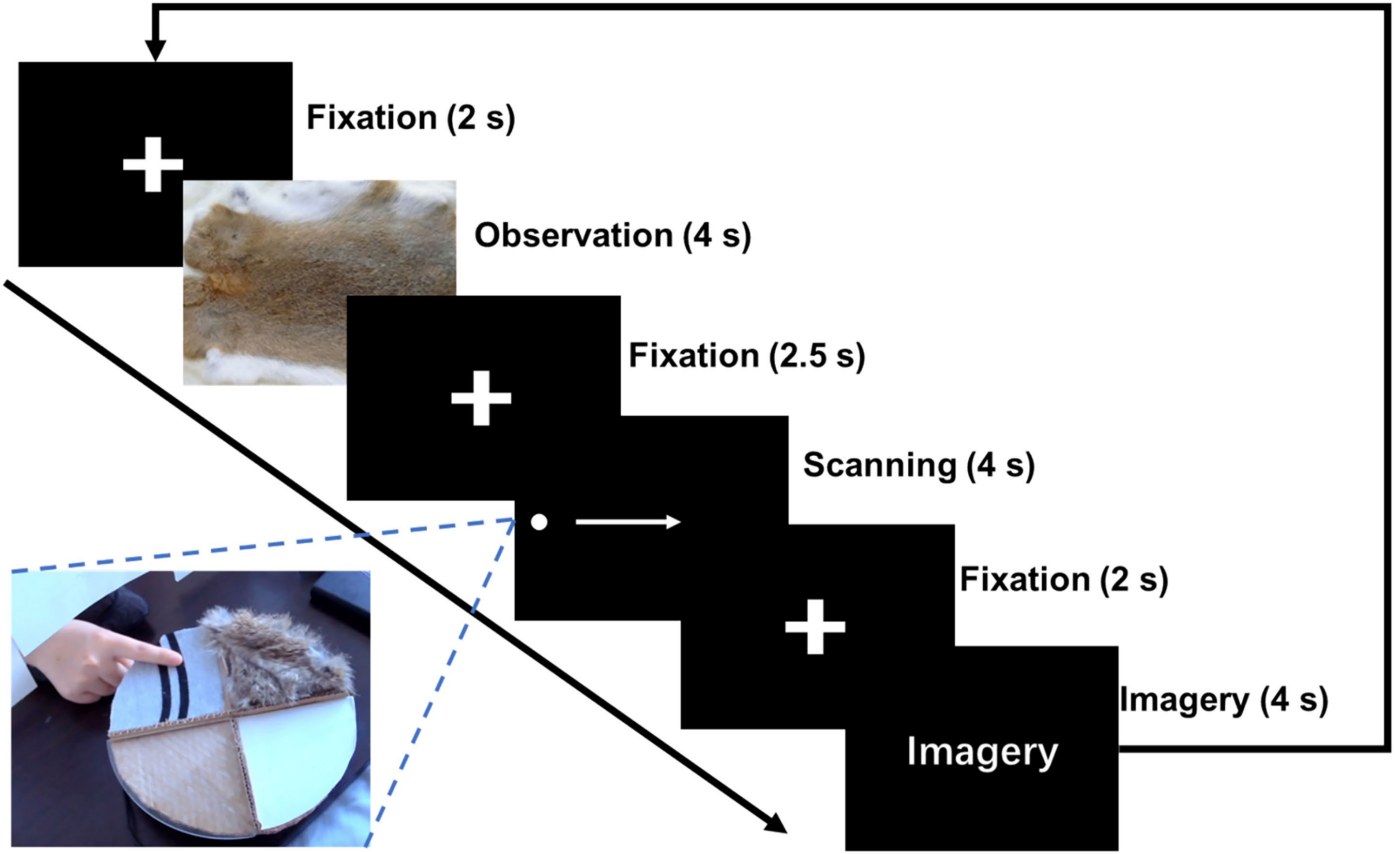
Experimental paradigm of texture cognitions. Three tasks of texture cognition were included in the experiment: Texture Observation (TO), Texture Scanning (TS), and Texture Imagery (TI).

The texture cognition task was structured into three distinct phases: texture observation (TO), texture scanning (TS), and texture imagery (TI, Figure 2). At the beginning of each trial, an image of the texture material was presented on the screen for 4 s. Following a 2.5-s interval, participants actively explored the corresponding physical texture from left to right using their index finger. The speed of finger moving was guided by a moving dot which traveled across the screen at a constant speed for 4 s. After a subsequent 2-s interval, participants were asked to imagine the tactile sensation of the texture they had just explored for another 4 s. The experiment comprised 10 blocks, with each block consisting of 20 entire trials covering TO, TS, and TI. The four materials were presented in a pseudo-randomized order within each block, ensuring that each block contained 5 trials per texture type.

### 2.4 Data recording and preprocessing

Neural activity was recorded by a 128-channel amplifier (Neuroscan, Australia) with a sampling rate of 1,000 Hz. For participants implanted with more than 128 recording contacts, priority was given to contacts located in the parietal cortex and other cortical gray matter regions. During data acquisition, channels exhibiting excessively great noise or impedance were manually excluded. To preprocess the EEG signals, a notch filter was applied to reduce 50 Hz power interference. Subsequently, a bandpass filter ranging from 2 to 90 Hz was implemented. To characterize the continuous neural dynamic across tasks, each epoch was extracted from −2 s to 16.5 s aligned to the onset of TO, which covered the entire task of TO, TS and TI. −2~0 s pre-TO period was used for baseline correction. Finally, the data were downsampled to 250 Hz for further analysis.

### 2.5 Temporal-spectral representation (TFR)

The Short-Time Fourier Transform (STFT) was applied to the entire trial (−2 to 16.5 s aligned to the onset of TO) to compute power in the time-frequency domain. To minimize carryover effects from preceding phases and anticipatory effects from subsequent phases, baseline power was defined as the power recorded during the period from −0.5 to −0.2 s before the onset of each task phase (TO, TS, and TI). Baseline correction was performed by division, followed by log transformation.

While the function of alpha and beta bands in tactile imagery has been discussed in EEG studies (Yakovlev et al., 2023; Yao et al., 2018), recent intracranial work has demonstrated that gamma-band (>30 Hz) activity exhibits great classification performance for tactile imagery (Bashford et al., 2021). Therefore, a broader range of frequency band (from delta to high gamma) was included to enable a comprehensive understanding of the spectral signatures underlying tactile perception. Average power was computed for six distinct bands: delta (2–4 Hz), theta (4–8 Hz), alpha (8–12 Hz), beta (12–30 Hz), gamma1 (30–60 Hz), and gamma2 (60–90 Hz). To account for variability in task onset times due to differences in reaction speed, TFR were analyzed using the average power recorded between 0.5 and 3.5 s following task initiation.

### 2.6 Classification

A shallow convolutional neural network (ShallowNet) was employed to classify the three tasks based on single-channel neural activities (Schirrmeister et al., 2017). For each task (TO, TS, and TI), band-filtered time-series sEEG signals recorded from 0 s to 4 s following task onset were used. The dataset was pooled across four texture conditions (50 trials per texture), resulting in 200 trials per task. The training set comprised 160 trials per task (480 trials in total), while both the validation and test sets included 20 trials per task (60 trials in total). This classification procedure was repeated for all six frequency bands.

### 2.7 Coherence

Coherence serves as a measure of functional connectivity by quantifying neural synchronization between brain regions within specific frequency bands. For each participant, pairwise coherence was computed between recording contacts in different parietal regions across all six frequency bands. A sliding window (2-s window with 100-ms step size) was employed to capture continuous coherence dynamics throughout the task period (−2 to 16.5 s relative to TO onset). To ensure comparability, coherence values were standardized to a standard deviation of 1 and baseline-corrected using the mean coherence from −2 to 0 s prior to the onset of each task phase (TO, TS, TI). Coherence analysis was performed using functions from the Chronux toolbox (Cold Spring Harbor Laboratory, NY, USA).

### 2.8 Granger causality

Granger-Geweke causality analysis was conducted to evaluate the directional information transfer among parietal regions (Dhamala et al., 2018). We applied conditional Granger causality (CGC) to assess the causal influence of one contact (i) on another (j) while accounting for the potential contributions of additional contacts (k). Within-participant CGC analysis was performed pairwise between recording contacts across different regions of interest. All contacts, except those from the same region as i, were designated as k, ensuring that their influence on j was accounted for in the analysis. The same sliding window approach (2-s window with 100-ms step size) and standardization procedure were applied to normalize CGC values.

### 2.9 Statistics

Non-parametric methods were employed in the current study due to small sample sizes in some tests. The Wilcoxon Signed-rank Test was used for comparisons for time-frequency representation and functional connectivity between results of the baseline and tactile processes, with the *p* value corrected for multiple comparison (e.g., p*3 when conducting multiple comparison between the baseline and TO/TS/TI). Two-sided tests were conducted as no clear hypothesis of the modulation direction was made. The Kruskal-Wallis test was used for classification accuracy among various frequency bands, with Bonferroni correction for post-hoc tests. All the statistical analyses were conducted in MATLAB 2020b (MathWorks, USA).

## 3 Results

### 3.1 Contrasting time-frequency representation for texture scanning and imagery

**Figure 3:**
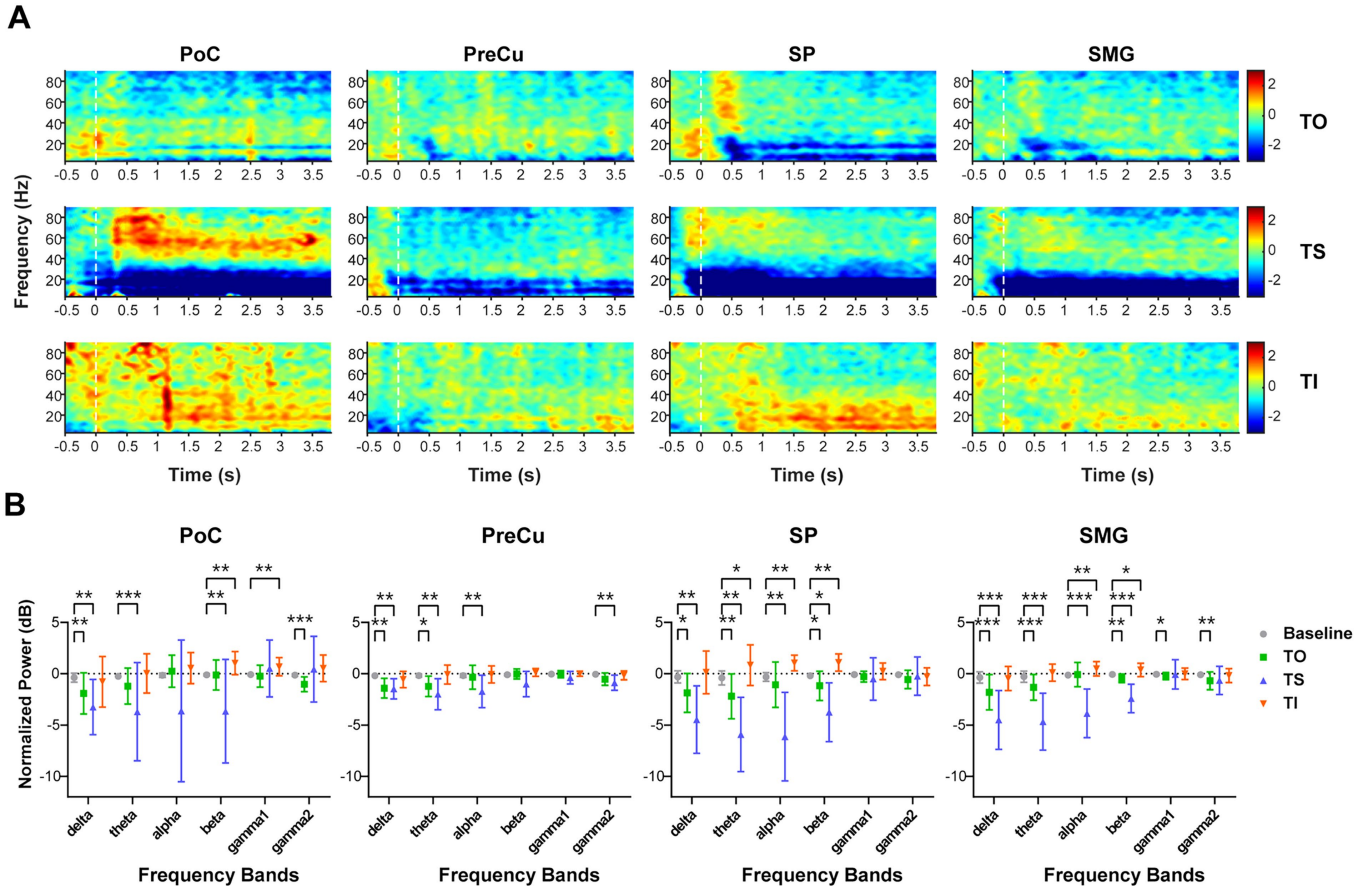
Time-frequency representation during texture processing. **(A)** Corrected time-frequency representation for the four brain regions at different task phases. The color map represents the calibrated power (dB). **(B)** Comparison of time-frequency representation between task and baseline for each frequency band. The Wilcoxon signed-rank test was used, and *p*-values were corrected for multiple comparisons. Sample sizes for each brain region were as follows: PoC (*n* = 17), PreCu (*n* = 12), SP (*n* = 12), SM (*n* = 24). *: *p* < 0.05; **: *p* < 0.01; ***: *p* < 0.001. Error bars represent standard deviation.

Firstly, we compared the local time-frequency representation for various tactile processes among parietal cortices. Across all four cortical regions of interest, power in the lower-frequency bands (below 30 Hz) exhibited significant decreases (ERD) during TS (Figures 3A,B). Weaker suppressions were observed in the delta and theta bands during TO, while the alpha band remained unaffected. In contrast, TI showed an overall increase of TFR relative to baseline. Specifically, power for alpha and beta bands significantly increased in SP and SMG, while beta and gamma1 power showed significant increases in PoC. No significant power changes were observed in PreCu compared to baseline. These findings indicated that while all three tactile processes modulated local neural encoding in parietal cortices including both the somatosensory and the PPC, they induced distinct patterns of TFR. The opposing responses observed between TS and TI suggest that these processes are governed by independent neural mechanisms.

**Figure 4:**
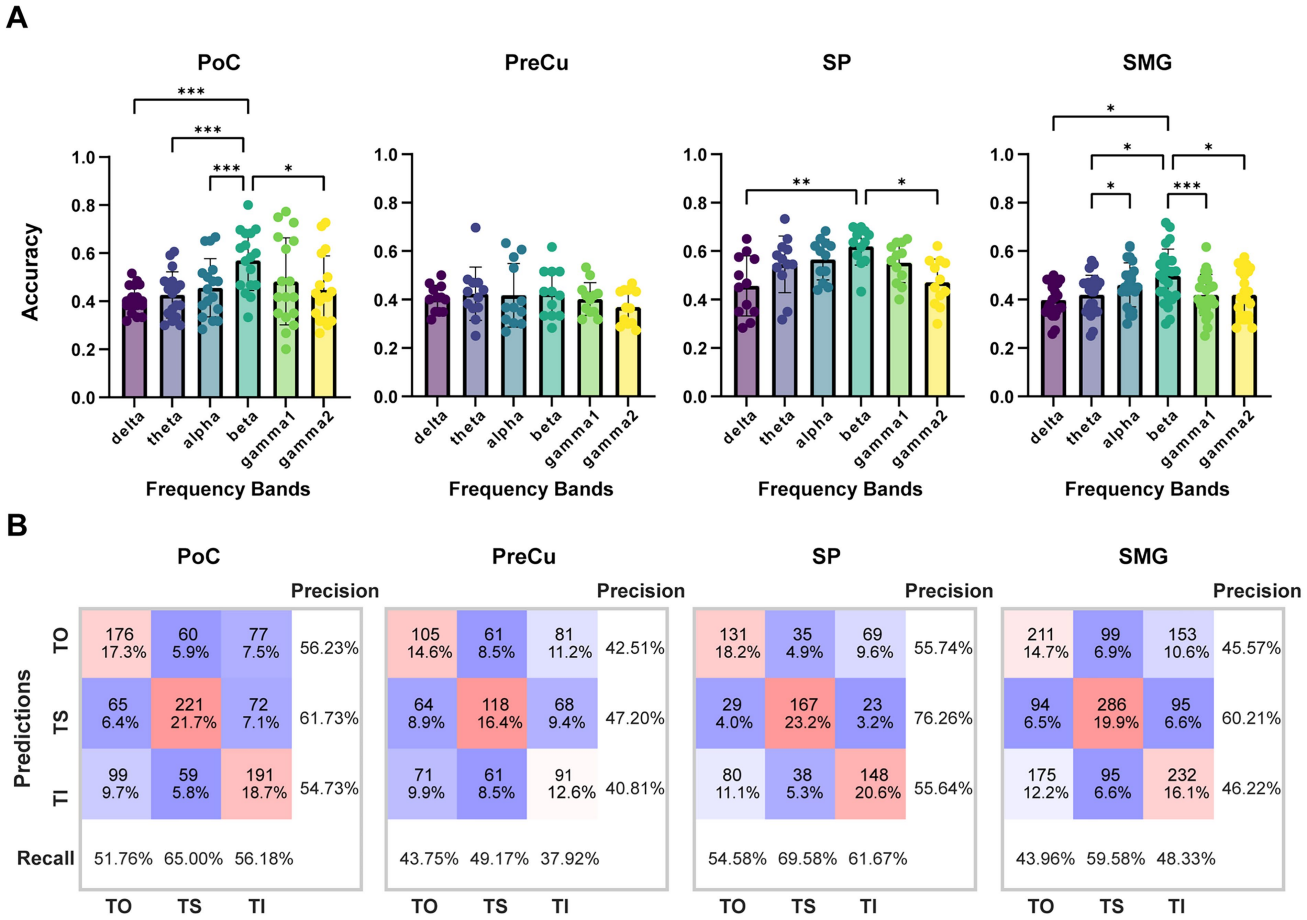
Classification accuracy of TO/TS/TI with single-contact data. **(A)** Classification accuracy across different frequency bands for each brain region. Sample sizes for each brain region are as follows: PoC (*n* = 17), PreCu (*n* = 12), SP (*n* = 12), SM *(n* = 24). Kruskal-Wallis test with Bonferroni correction was applied for comparison between frequency bands, *: *p* < 0.05; **: *p* < 0.01; ***: *p* < 0.001. Error bars represent standard deviation. **(B)** Confusion matrix of classification for beta band. The counts represent the total number of samples for all contacts in the test set, which is n*60 (60 trials in the test set).

To further validate task-related differences, ShallowNet was employed to classify TO, TS, and TI using single-channel sEEG data. As a result, classification accuracy for each brain region exceeded chance levels (Figure 4A). In PoC, SP, and SMG, the beta band revealed the highest classification accuracy (mean ± std.: PoC: 56.96 ± 12.51%, SP: 61.94 ± 7.77%, SMG: 49.75 ± 11.09%), highlighting the importance of beta band features in texture processing. The confusion matrix of beta band revealed the greatest precision for TS in all brain regions, indicating that TS achieved the greatest effect on parietal neural activities, while neural responses for TO and TI were weaker and were more likely to be confused against each other (Figure 4B).

### 3.2 Interregional coherence is frequency-related during texture processing

**Figure 5:**
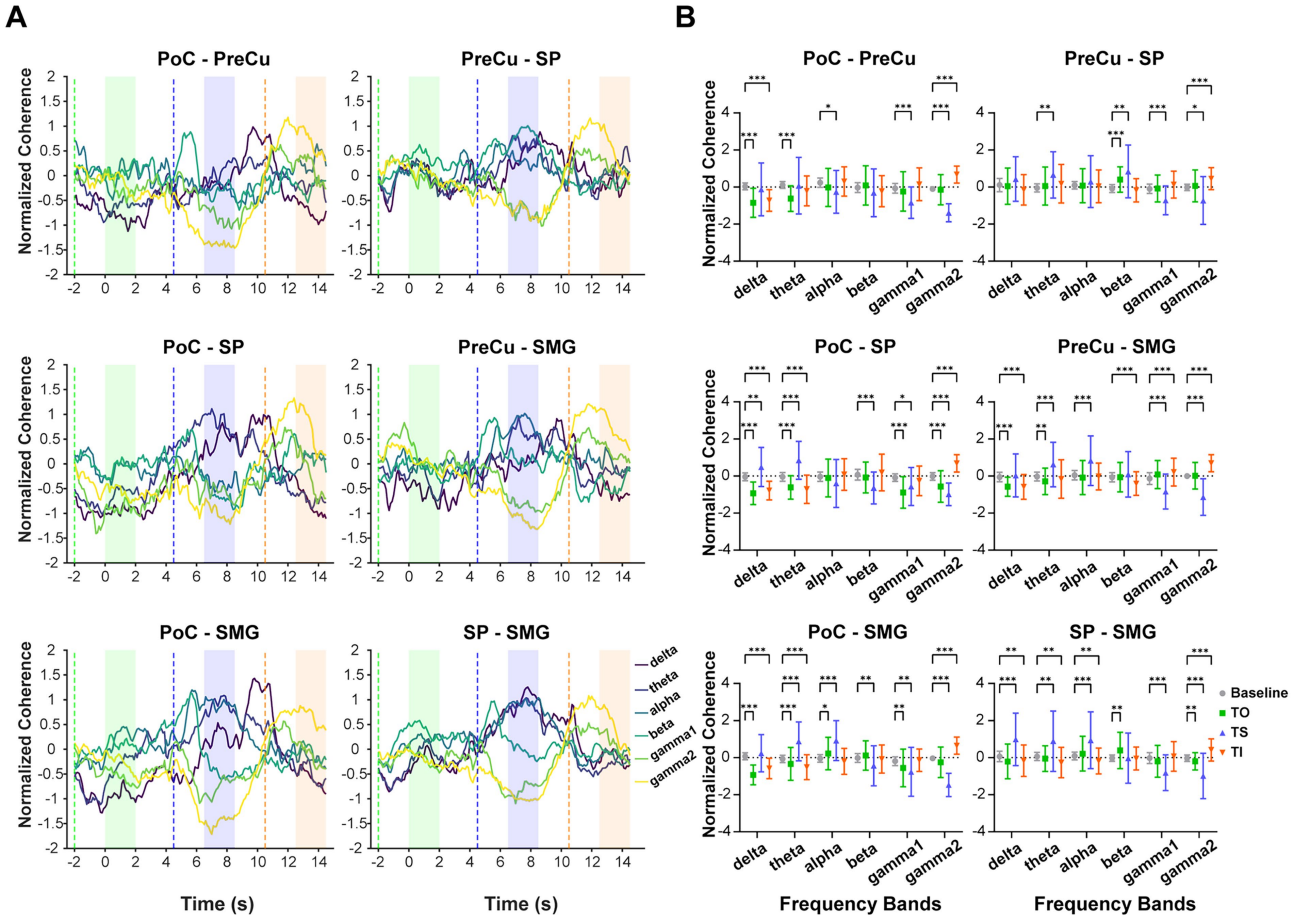
Coherence across texture processing tasks and frequency bands. **(A)** The mean standardized coherence between each pair of brain regions over time. Coherence was calculated using a 2-s window with a 100-ms sliding step. The x-axis represents the time corresponding to the left edge of the window, aligned with the onset of TO task at 0 s. The dashed line indicates when the moving window starts entering the task phase, while in the shaded area the moving window is fully covered by the task phase. Colors of shaded areas match with tasks: TO: green; TS: blue; TI: orange. **(B)** Standardized coherence compared between tasks and baseline. The sample sizes for coherence between brain regions are as follows: PoC-PreCu: *n* = 36 pairs of contacts, PoC-SP: *n* = 46, PoC-SMG: *n* = 78, PreCu-SP: *n* = 41, PreCu-SMG: *n* = 72, SP-SMG: *n* = 59. Wilcoxon Signed-rank test with adjusted *p*-values was applied for multiple comparisons. *: *p* < 0.05; **: *p* < 0.01; ***: *p* < 0.001. Error bars represent standard deviation.

Coherence analysis was conducted to characterize interregional synchronization patterns, providing insights into neural communication within the parietal cortex. The coherence dynamics revealed a frequency-dependent modulation. During TS, coherence increased in lower-frequency bands (below 8 Hz) but decreased in higher-frequency bands (above 30 Hz). Conversely, during TI, coherence decreased in lower-frequency bands but increased in higher-frequency bands. In consistent with TFR, results of coherence confirmed the distinct neural dynamics between texture scanning and imagery from the perspective of interregional communication, in which tactile processes were potentially interacted with frequency. Notably, gamma2 coherence (60–90 Hz) showed significant deviations from baseline during both TS and TI across all pairs of parietal regions, suggesting that high-frequency interregional synchronization plays a critical role in both the perception and the reconstruction of textural sensations (Figure 5).

### 3.3 Unique neural communication patterns for texture scanning and imagery

**Figure 6:**
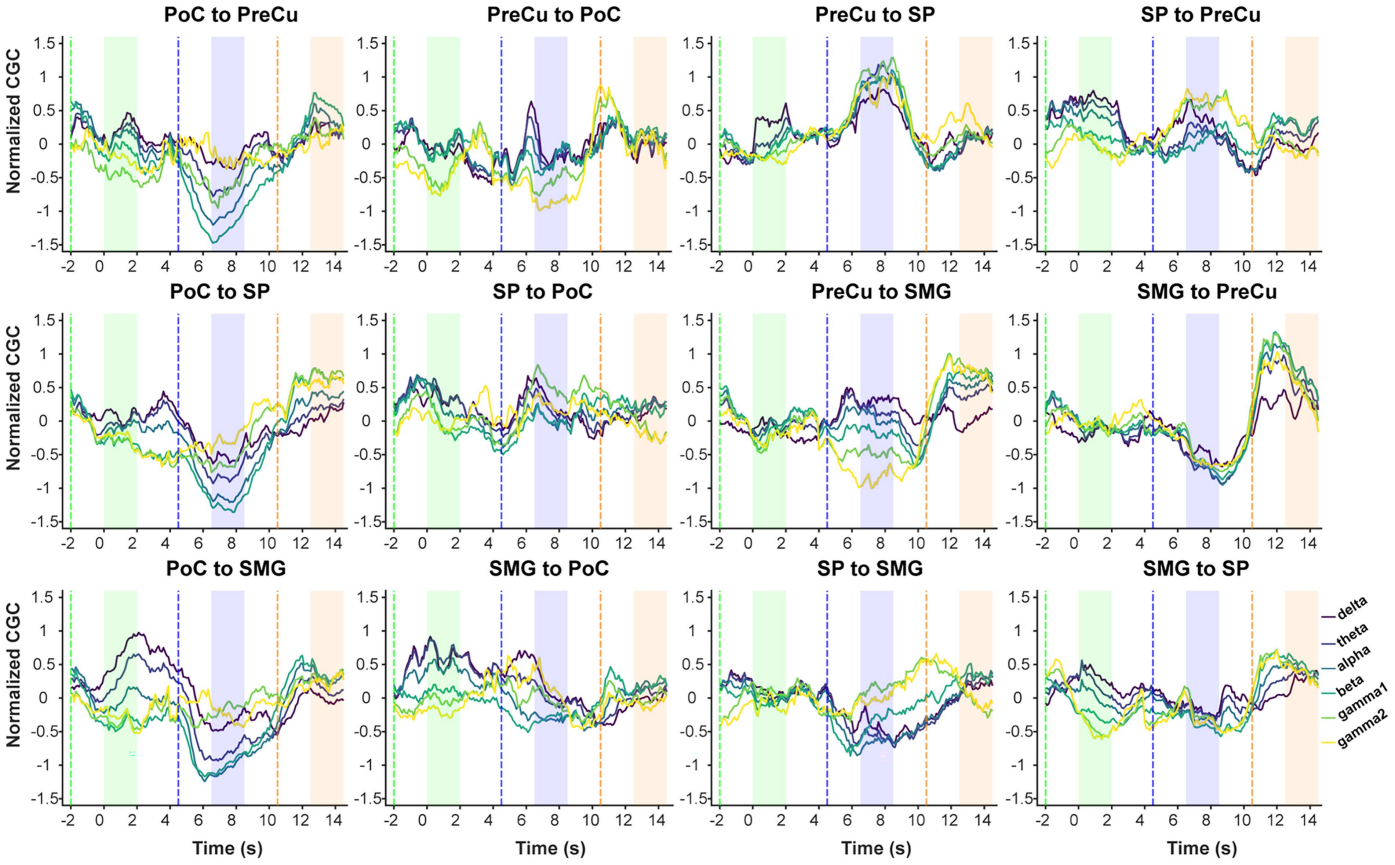
Continuous conditional Granger causality. Temporal dynamics of mean standardized CGC. CGC was computed using a 2-s sliding window with a 100-ms step. The x-axis represents the time corresponding to the left edge of the window, with 0 s aligned to the onset of the texture observation task. Dashed lines indicate the time when the sliding window entered the task phase, while in the shaded area the moving window is fully covered by the task phase. Colors of shaded areas match with tasks: TO: green; TS: blue; TI: orange.

**Figure 7:**
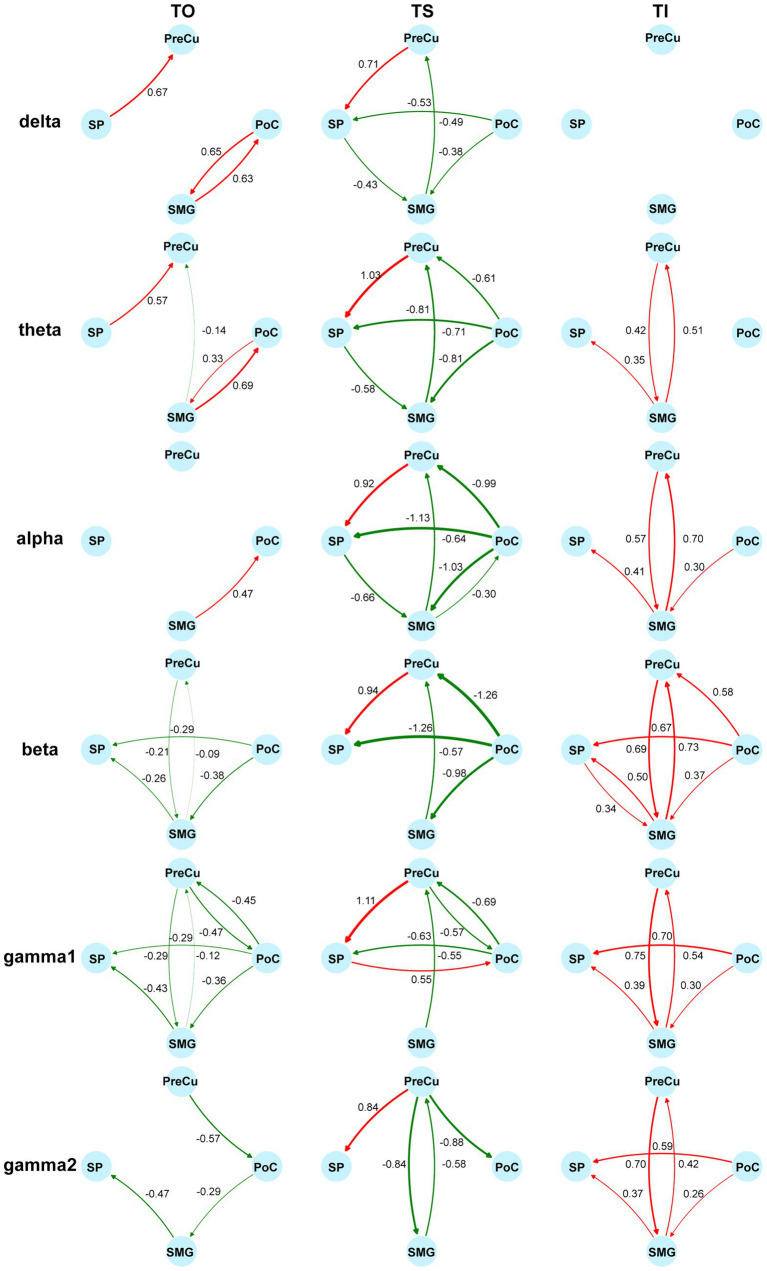
Modulation of directional information flow in the parietal network. Information flow is quantified by the difference of standardized CGC between tasks and the baseline. The columns reflect tactile processes (TO/TS/TI) and the rows reflect bands. Increases of CGCs are colored in red and decreases of CGCs are colored in green. Non-significant differences from the baseline are not shown (Wilcoxon signed-rank test, adjusted *p* < 0.05). Sample sizes are as follows: PoC-PreCu: *n* = 36, PoC-SP: *n* = 46, PoC-SMG: *n* = 78, PreCu-SP: *n* = 41, PreCu-SMG: *n* = 72, SP-SMG: *n* = 59, for both directions.

Furthermore, conditional Granger causality analysis was conducted to investigate the directional information flow within the parietal cortical network (Figure 1B). Firstly, bidirectional CGC between the somatosensory cortex and posterior parietal cortices were compared between texture scanning and imagery. During TS, CGCs from PoC to PPC regions were reduced compared to baseline, particularly in the alpha and beta frequency bands (Figures 6, 7), indicating that TS suppressed somatosensory-to-PPC neural communication. In contrast, during TI, CGCs from PoC to SP and SMG were enhanced in the beta and higher frequency bands, suggesting a dissociable dynamics in neural communication from the somatosensory cortex to PPC between TS and TI. In terms of projections from PPC to the somatosensory cortex, TS facilitated gamma1 CGC from SP to PoC while inhibiting gamma1 and gamma2 CGCs from PreCu to PoC. However, no significant modulation of CGC was observed from PPC regions to PoC during TI, suggesting the pathway from the somatosensory cortex to the posterior parietal cortex is unidirectional during the reconstruction of tactile sensation.

We then compared CGCs within PPC regions to identify the effect of tactile processes on intra-PPC communications. Across all frequency bands, TS was found to enhance CGCs from PreCu to SP but inhibit CGCs from SMG to PreCu. Conversely, TI facilitated bidirectional communication between SMG and PreCu. In fact, TI achieved an overall increase in intra-PPC CGCs, but not in CGCs from PreCu to SP. The results indicated that the reconstruction of tactile sensation activated neural communication within the PPC in a pattern completely different to tactile exploration. Together, our finding highlighted the uniqueness of functional parietal networks during texture scanning and imagery.

## 4 Discussion

The current study identified distinct patterns of neural activity and communication in the parietal cortex during texture cognitions with invasive sEEG. Texture scanning induced ERD in the low-to-mid-frequency bands of the somatosensory and posterior parietal cortices, whereas texture imagery induced ERS. Frequency-dependent interregional synchronization was observed, with opposing trends in low- and high-frequency bands. Additionally, texture scanning suppressed neural communication from the somatosensory cortex to the PPC, whereas imagery enhanced both somatosensory-to-PPC and intra-PPC communication. These findings reveal unique local and network-level neural dynamics underlying tactile perception and reconstruction. Our work shed light on the neural mechanism underlying the formation of tactile experience, providing theoretical foundation for inducing biomimetic artificial sensations in patients with paralysis.

### 4.1 Local neural encoding for texture processing in parietal cortices

Neuroimaging studies suggested that tactile perception and imagery share a common neural substrate, leading to activations in the somatosensory cortex during tactile imagery (Schmidt et al., 2014; Schmidt and Blankenburg, 2019; Yoo et al., 2003). This is also supported by electrophysiological evidence, as ERD was observed in both tactile stimulation and imagery (Wen et al., 2024; Yakovlev et al., 2023). Consistent with previous findings (Henderson et al., 2022), we found that texture scanning suppressed neural oscillations below 30 Hz in the parietal cortices. However, texture imagery did not induce ERD but instead induced ERS. These results indicated distinct patterns of neural activities despite overlapped cortical locations. Limited by low spatial resolution, previous EEG studies primarily focused on the time-frequency representation of the central region, which integrated neural activity from multiple sources, including the precentral and postcentral gyri. ERS in the somatosensory cortex might be difficult to detect due to the great ERD in the motor cortex during the imagery. Though not explicitly stated, some studies observed alpha-beta ERS in the posterior parietal and occipital regions during sensory imagery (Sengupta and Lakshminarayanan, 2024; Yakovlev et al., 2023; Yao et al., 2018), suggesting that ERS in PPC and somatosensory cortex might have been overlooked. Unlike EEG studies, we used invasive sEEG to precisely record intracortical neural activity, enabling more accurate localization of cortical representations during texture imagery.

Alpha and beta desynchronization have traditionally been considered key signatures of sensorimotor processing (Engel and Fries, 2010; Sigala et al., 2014). In the somatosensory cortex, power in both alpha (Klimesch, 2012; Su et al., 2020) and beta (Cheyne et al., 2003; Gaetz and Cheyne, 2006; Kilavik et al., 2013) bands is typically suppressed during tactile stimulation or sensorimotor coordination. This suppression is thought to reflect increased cortical excitability, thereby facilitating the processing of cutaneous and proprioceptive inputs (Gaetz and Cheyne, 2006). Alternatively, ERS is often interpreted as a marker of local neural inhibition (Klimesch, 2012; Neuper et al., 2006). In light of this framework, the ERS observed in our study may indicate a suppression of bottom-up sensory input during the imagery process, thereby allowing attention to be directed more effectively toward endogenously generated perceptual experiences. Another possibility is that the observed ERS may reflect working memory processes. The tactile imagery task in our experiment essentially involved the mental reconstruction of a previously experienced sensation, which engages working memory—a function closely linked to beta oscillations (Engel and Fries, 2010; Spitzer and Haegens, 2017). Numerous studies have demonstrated that working memory engagement is associated with increased beta-band power (Lundqvist et al., 2016, 2018; Schmidt et al., 2019). Thus, the observed increase in beta power likely reflects working memory processing. Similarly, recent findings suggest that alpha-band ERS can occur during the retention phase of working memory tasks (Wianda and Ross, 2019; Zhozhikashvili et al., 2022). In summary, our results indicated that while both the somatosensory and posterior parietal cortices are involved in texture scanning and imagery, the neural synchronization patterns underlying these processes are distinct.

### 4.2 High-gamma interregional synchronization distinguishes texture scanning and imagery

We investigated the communication between the somatosensory cortex and PPC (SP, PreCu, SMG) through coherence analysis, based on the principle that effective communication occurs between phase-locked oscillations, ensuring synchronous input–output coupling (Fries, 2005). Consistent with the time-frequency representation, interregional synchronization revealed contrasting patterns between texture scanning and imagery. Notably, this coherence was frequency-dependent, indicating an interaction between tasks (scanning vs. imagery) and oscillatory frequency. Our findings highlighted the role of high-frequency gamma coherence (>60 Hz) in texture scanning and imagery. Gamma activity has been recognized as a fundamental mechanism underlying cortical information processing (Fries, 2009) and has been implicated in tactile perception (Ryun et al., 2017). Interregional gamma synchronization is known to modulate the efficacy, precision, and selectivity of neural communication (Buzsáki and Schomburg, 2015; Fries, 2005, 2015). Our results revealed a suppression of high-frequency communication across parietal regions during texture scanning, whereas the communication was enhanced during imagery. This enhancement was likely driven by selective attentions, as tactile imagery directed attentional resources toward the fingers (Ahn et al., 2014; Yao et al., 2019), a top-down process known to strengthen gamma coherence (Bauer et al., 2006; Vinck et al., 2013). The pattern of gamma2 interregional communication is also supported by results of Granger causality, which demonstrated a significant enhancement of gamma2 information flow during imagery. Collectively, these findings suggested that high-gamma interregional communication obtains distinct task-dependent patterns, with a pronounced activation during top-down processing.

### 4.3 Opposing modulation of parietal communication by tactile perception and reconstruction

The posterior parietal cortex is well established as a high-level sensory region that integrates sensory inputs and modulates sensorimotor interactions (Freedman and Ibos, 2018; Whitlock, 2017). Prior research has demonstrated that specific subregions, such as the SMG and the intraparietal sulcus (IPS), are involved not only in responding to tactile stimuli but also in tactile imagery and observation (Bashford et al., 2021; Chivukula et al., 2021, 2025). One step further, our findings revealed that the PPC, particularly the SMG and SP, plays a critical role in texture processing, including both observation and imagery.

Our study further investigated the long-range projection within the parietal cortex. Contrary to the expected bottom-up sensory flow, we observed suppressed causal relationship from the somatosensory cortex to the posterior parietal cortex. The suppression was possibly attributed to movement-related sensory gating, that texture-related neural projection was inhibited by finger movements (Chapin and Woodward, 1982; Song and Francis, 2015; Urbain and Deschênes, 2007). The finding suggested that PPC, in coordinating motor functions, actively suppressed the bottom-up projection of sensory information. During imagery, projection from the somatosensory cortex to the motor cortex was enhanced above 30 Hz, indicating that even in the absence of actual sensory input, the somatosensory cortex continued to encode and transfer tactile information to higher-order centers. Besides, although the causal relationships revealed dissociable trends between scanning and imagery from PoC to PPC, no modulation on the opposite information flow (from PPC to PoC) was observed during imagery. Thus, our results did not support the hypothesis that imagery converses the direction of information flow of tactile perception (Dentico et al., 2014). Instead, our findings suggested that texture perception and imagery exerted a unidirectional influence on bottom-up projection, with top-down neural communication unaffected by texture cognitions.

Furthermore, we found that the internal network within PPC was actively engaged during texture imagery. Notably, bidirectional information transfer between the SMG and PreCu was significantly enhanced across frequency bands above 4 Hz. The SMG has been implicated in both tactile observation and imagery (Bashford et al., 2021), while the PreCu is known to contribute to imagery tasks (Cabbai et al., 2024; Schmidt et al., 2014; Tomasino et al., 2022). Moreover, the PreCu is considered a key component of the core construction network, which is engaged in cognitive processes such as recalling past experiences or imagining unreal events (Dadario and Sughrue, 2023; Hassabis and Maguire, 2009; Madore et al., 2016; Spreng et al., 2009; Summerfield et al., 2010; Yamaguchi and Jitsuishi, 2024). Our results suggested that the SMG-PreCu pathway is crucial in texture-related tactile imagery. The distinct modulation of observation and imagery on SMG-PreCu pathway indicated its specific involvement in the active reconstruction of past experiences rather than in the passive recognition of texture concepts.

### 4.4 Limitation and future direction

Due to the limited time available for patient participation, our study did not include a passive texture stimulation condition, which could further clarify the influence of movement on sensory processing, thereby providing a clearer theoretical framework for texture-related neural projection. Future research should aim to compare texture perception under conditions with and without movement, as well as examine differences in the parietal network between passive texture perception and active tactile imagery.

## Data Availability

The datasets presented in this article are not readily available because the datasets generated in the current study are not open to public due to data privacy regulations of patient data. Requests to access the datasets should be directed to Changyong Wang, wcy2000_zm@163.com.

## Footnotes

^1^ www.freesurfer.net/

^2^ www.fil.ion.ucl.ac.uk/spm/
